# The relationship between trait mindfulness and inclusive education attitudes of primary school teachers: a multiple mediating model

**DOI:** 10.3389/fpsyg.2023.1280352

**Published:** 2023-12-20

**Authors:** Fenxia Huang

**Affiliations:** Basic Education College, Putian University, Putian, China

**Keywords:** primary school teachers, trait mindfulness, empathy, teacher efficacy for inclusive practice, inclusive education attitudes, inclusive education

## Abstract

**Objective:**

This study aims to explore the impact of primary school teachers’ empathy and efficacy for inclusive practice on the relationship between trait mindfulness and inclusive educational attitudes.

**Methods:**

A total of 606 primary school teachers were tested using the Five Facet Mindfulness Questionnaire, Interpersonal Response Index Scale, Teacher Self-efficacy for Inclusive Practice Scale, and Teachers’ Multidimensional Attitudes toward Inclusive Education Scale.

**Results:**

Primary school teachers’ trait mindfulness is significantly positively correlated with inclusive educational attitudes. Primary school teachers’ trait mindfulness has an indirect impact on inclusive education attitudes through empathy, and primary school teachers’ trait mindfulness has an indirect impact on inclusive education attitudes through teacher efficacy for inclusive practice. In addition, empathy and teacher efficacy for inclusive practice play a sequential mediating role between primary school teachers’ trait mindfulness and inclusive education attitudes.

**Conclusion:**

This empirical study reveals that empathy and efficacy for inclusive practice play a sequential mediating role between primary school teachers’ trait mindfulness and inclusive education attitudes. On one hand, this research contributes to enriching the outcomes in the field of inclusive education for primary school teachers, providing a theoretical foundation for the study of their inclusive education attitudes. On the other hand, the study offers a detailed explanation of the psychological mechanisms behind the impact of mindfulness traits on the inclusive education attitude of primary school teachers, guiding schools in implementing mindfulness-based intervention programs.

## 1 Introduction

In the context of mainstream education, inclusive education for special-needs students has become a global movement, making significant contributions to providing equal education opportunities and facilitating convenient learning and living conditions for a vast population of disadvantaged individuals (Lindner and Schwab, 2020). This holds paramount significance in promoting educational equity, balance, and sustainable development. Therefore, inclusive education has emerged as a crucial trend in global educational reforms. Despite the strong political advocacy for inclusive education, its successful implementation largely depends on teachers (Pit-ten Cate et al., 2018). However, research indicates that not all teachers embrace the principles of inclusive education, and some hold negative attitudes, being unwilling to accept students with special education needs (Monsen et al., 2014).

Numerous factors can influence teachers’ attitudes and willingness to engage in inclusive education (Štemberger and Kiswarday, 2018). For instance, research by Moberg et al. (2020) suggests that teachers’ positive attitudes toward inclusive education depend on successful experiences in its implementation rather than mere practical exposure. Savolainen et al. (2022) mention in their study that teachers harbor various concerns about the implementation of inclusive education, particularly when dealing with challenging student behavior, as they may feel inadequately equipped to address such challenges. According to Mngo and Mngo (2018), teachers perceive their training in special education and inclusive education stages as insufficient to ensure the smooth integration of disabled students into regular classrooms. Therefore, the support and management provided by schools may be crucial factors influencing teachers’ attitudes towards inclusive education.

Inclusiveness in education is critical to the development of abilities, mental health, and social adjustment of students with special needs (Noreen et al., 2019). At the same time, it is also an effective way for the country to build an inclusive society and realize education for all. The fundamental pillars of inclusive education are teachers, and teachers’ attitudes toward inclusive education strongly influence the extent to which teachers implement inclusive practices (Pit-ten Cate et al., 2018). Therefore, we must understand the factors related to teachers’ attitudes and willingness to inclusive education in order to better promote inclusive practice. As a form of metacognition, mindfulness not only enables individuals to pay attention to their own experiences and achieve self-calm, but also enables individuals to influence people’s consciousness in conjunction with the wider world and cultural patterns. This allows mindfulness to combine ethics, compassion, and an appreciation for life itself, deepening one’s awareness of social inclusion, and ultimately situating oneself in the context of how one can contribute to others and to the sustainable development of society (Goldman Schuyler et al., 2021). According to the Organizational Model of empathy (Davis, 1996), a typical empathic episode is envisioned as the observer making contact with the target in some way, after which the observer generates cognitive, emotional, and/or behavioral responses. Mindfulness, as a source of positive emotions (Garland and Fredrickson, 2013), can cultivate teachers’ self-awareness and empathy (Jones et al., 2019). This enables teachers to attentively address students’ needs and better cope with the challenges of inclusive education, fostering a more positive attitude towards inclusive education. Therefore, this study will explore the impact mechanism of mindfulness on the attitudes of primary school teachers towards inclusive education, hoping that through this research, we can further improve primary school teachers’ attitudes towards inclusive education and encourage their active participation in inclusive education practices.

## 2 Literature review and theoretical hypotheses

### 2.1 Trait mindfulness and inclusive educational attitude

Mindfulness originated from Buddhist meditation and is often described as a naturally occurring ability that enables individuals to pay non-judgmental attention to present experiences (Kabat-Zinn and Hanh, 2009). Mindfulness can manifest as both a state and a trait. TM refers to a non-habitual, non-judgmental way of thinking that individuals establish, maintaining an objective stance to experience the general trend of the present moment (Brown and Ryan, 2003). TM is associated with various adaptive functions in individuals, such as attention control, psychological health, and reduction of pain and anxiety (Klainin-Yobas et al., 2016). For example, evidence from (Hwang et al., 2017) suggests that TM can enhance teachers’ sense of well-being and improve their ability to cope with high-pressure demands and emotional needs in teaching.

Saloviita (2020) points out that the successful implementation of inclusive education largely depends on teachers’ attitudes toward it. Attitude encompasses cognitive, emotional, and behavioral components, influencing individuals’ judgments and guiding social behavior. In this context, cognition refers to teachers’ views and concepts regarding inclusive education, emotion pertains to teachers’ emotional experiences related to inclusive education, and behavioral intention refers to teachers’ responses or tendencies in relation to inclusive education (Pit-ten Cate et al., 2018). Individual intentions and attitudes toward a particular event determine their behavior (Avramidis et al., 2019). Therefore, teachers’ attitudes may indeed have an impact on their willingness and effort in implementing inclusive education.

Despite significant efforts by various countries to implement inclusive education, and the general agreement among most teachers, implementing inclusion in mainstream classrooms remains a significant challenge for educators. For example, Van Mieghem et al. (2020) mention that teachers must adjust teaching methods and pace according to students’ special needs while maintaining high overall class performance and engaging in research activities. Crispel and Kasperski (2021) also indicate that although teachers generally have a positive attitude towards inclusive education, they encounter numerous challenges in practice due to a lack of specialized knowledge in special education and teaching methods. Over time, teachers may become concerned about their ability to successfully implement inclusive education and develop a negative attitude. However, there is reason to believe that TM has a positive role in inclusive education. For instance, research by Lavy and Berkovich-Ohana (2020) suggests that TM effectively alleviates teacher fatigue and stress, regulates emotional responses, and enhances the ability to cope with challenges, thereby reducing the pressure on teachers in inclusive education. Kalafatoğlu and Turgut (2019), in their study on mindfulness, also point out that TM may influence teachers’ ability to care for students, which is a crucial factor in inclusive education. Therefore, we believe that under the positive influence of TM, teachers’ attitudes toward inclusive education can undergo significant positive changes.

### 2.2 TM, empathy, and inclusive education attitudes

Empathy, as an essential ability, has drawn attention from researchers. Empathy is the ability to recognize and share the emotional experiences of others, constituting a positive emotional response. Empathy consists of cognitive and emotional components. Cognitive empathy refers to an individual’s ability to adopt others’ perspectives, understand others’ emotional experiences, and identify these emotional experiences (Blair, 2005). Emotional empathy refers to an individual’s emotional response to others’ emotional experiences, such as sensitivity to others’ emotions and sharing others’ emotional experiences (Reniers et al., 2011). According to Wink et al. (2021), teachers with empathy can empathize with students’ thoughts and feelings from their perspective, adjusting their teaching methods to better facilitate students’ growth. Especially for students with special needs, teachers’ empathy appears to play a crucial role.

TM involves the ability to perceive and recognize one’s own emotional states, and during interpersonal communication, it enhances attention to and understanding of emotional cues from others (Brown and Ryan, 2003). Some researchers suggest that mindfulness interventions can be an effective means to cultivate and improve empathy (Bellosta Batalla et al., 2017). For example, conclusions from neuroscience studies indicate that mindfulness meditation can promote an increase in gray matter volume or density in the brain, thereby enhancing an individual’s self-awareness, emotional regulation, and attention control (Tang et al., 2020). This, to some extent, facilitates an individual’s understanding of others’ thoughts, cognition, and emotions, which are core elements of empathy. Furthermore, Bibeau et al. (2020) point out that individuals, through mindfulness training, can increase opportunities for prosocial behavior and emotion by promoting real-time awareness of others’ suffering, thereby enhancing empathy. Therefore, mindfulness may be a key factor in developing empathy.

In the educational context, empathy is considered a predictive factor for teachers’ positive behavior, especially in inclusive education, where teachers’ empathy plays a crucial role. Inclusive teaching requires that teachers’ educational philosophies and methods meet the individual and collective needs of all students. Moreover, for teachers to create an inclusive and fair atmosphere in the classroom, teacher empathy is a prerequisite (Makoelle, 2019). Navarro-Mateu et al. (2019) point out that empathy can increase teachers’ understanding and care for special-needs students, influencing their attitudes towards them and ultimately shaping their attitudes toward inclusive education. Therefore, teachers with a high level of empathy not only psychologically endorse inclusive education but also, in educational practice, understand the difficulties and needs of students from their perspective. This allows students to feel understood and recognized, and teachers can effectively provide support and assistance in both academic and life aspects (Makoelle, 2019).

### 2.3 TM, teacher efficacy for inclusive practice and inclusive educational attitude

Self-efficacy refers to an individual’s confidence in their ability to utilize acquired skills to perform a specific job or behavior (Bandura, 1977). This theory has been widely applied in the research field of inclusive education. Numerous studies indicate that teachers’ levels of self-efficacy play a crucial role in implementing inclusive practices (Ahsan et al., 2013; Sharma et al., 2013). Self-efficacy not only influences teachers’ instructional decisions and inclusive education behaviors but is also linked to the concepts, actions, and persistence involved in helping all students succeed (Cardona-Molto et al., 2020). Loreman et al. (2013) suggest that, in the process of implementing inclusive education, teachers’ confidence in their knowledge, skills, and abilities is a key factor for successful implementation. Moreover, teachers with high self-efficacy are more likely to accept students with behavioral issues in regular classrooms and are willing to provide them with differentiated learning services (Soodak and Podell, 1993; Weiss et al., 2019). Therefore, teachers’ self-efficacy in inclusive education is considered a crucial factor influencing the implementation of inclusive teaching.

According to the Job Demands-Resources (JD-R) model (Demerouti et al., 2001), individuals are prone to overwork and experience symptoms of fatigue when facing high job demands and limited resources. For instance, Hopman et al. (2018) pointed out that teachers often feel emotionally exhausted when dealing with children with highly disruptive behavior over the long term. Although teachers’ self-efficacy can moderate these stresses and fatigue (Putwain and von der Embse, 2019), the recurring emotional events have a more significant impact on individuals’ emotions, work motivation, and well-being than singular major events (Lazarus and Folkman, 1987). Noetel et al. (2019) propose that mindfulness training can help individuals eliminate negative cognitions, alleviate the impact of emotions on work, shift attention to beneficial and task-related situations, and sustain individuals’ confidence and enthusiasm for their work, thereby enhancing self-efficacy levels in the long run.

Teachers’ attitudes toward inclusive education seem to be related to their inclusive efficacy (Sharma et al., 2015; Woodcock et al., 2022). For instance, Özokcu (2018) research found that teachers with higher self-efficacy tend to have more positive attitudes toward inclusive education and students with special needs. Savolainen et al. (2022) also noted that teachers’ self-efficacy contributes to cultivating their inclusive attitudes and willingness to implement inclusive education. The reason behind this is that teachers with higher self-efficacy levels have confidence in successfully implementing inclusive education. Moreover, in practical terms, teachers with high self-efficacy are resourceful and can employ differentiated teaching strategies to support all students, thereby better promoting their development (Breyer et al., 2020). Therefore, teacher efficacy for inclusive practice is likely to have a positive impact on their attitudes toward inclusive education.

### 2.4 Empathy and teacher efficacy for inclusive practice

The teacher’s empathy is crucial for inclusive teaching practices. With the implementation of inclusive education, the diversity of students increases. Teachers are not only required to provide an equitable education that meets the needs of all students but also to ensure that the education provided is genuinely beneficial to the students. If teachers lack empathy, the extent to which they can achieve this is questionable (Makoelle, 2019). Teacher self-efficacy is the belief that teachers have the capability to take necessary actions to successfully complete specific teaching tasks. It is a belief in one’s own abilities. Teacher self-efficacy involves judgments, beliefs, and feelings about educational values and personal teaching capabilities, significantly influencing teachers’ specific behavioral performances (Tschannen-Moran et al., 1998; Weiss et al., 2019).

Inclusive education aims to focus on students’ strengths, rejecting the labeling and isolation of students based on cognitive impairments and behavioral issues. Moreover, when teachers understand their relationship with both themselves and special students, they can emotionally comprehend the students’ inner worlds, thereby creating a trusting relationship and meaningful learning experiences for the students. Therefore, the ability of teachers to empathize, to consider things from the students’ perspective, is crucial for inclusive teaching (Goroshit and Hen, 2016). Teachers with empathy find a strengthened sense of purpose in their profession, allowing them to sustain the motivation and source of their self-efficacy (Bellah, 2007). Therefore, empathy also plays a driving role in their self-efficacy.

### 2.5 Current research

While research has already focused on teachers’ inclusive education attitudes, there is currently limited research on the relevant factors and mechanisms influencing these attitudes. This study aims to explore the relationship between TM and primary school teachers’ inclusive education attitudes from a metacognitive perspective, as well as the mechanisms between them. The main questions addressed in this study are: (1) Is TM related to inclusive education attitudes? (2) Does empathy mediate the relationship between TM and inclusive education attitudes? (3) Does teacher efficacy for inclusive practice mediate the relationship between TM and inclusive education attitudes? (4) Do empathy and teacher efficacy for inclusive practice have multiple mediating effects between TM and inclusive education attitudes?

Current Research Based on the above literature review and theoretical basis, the following hypotheses are proposed:

*Hypothesis 1*: TM is significantly positively correlated with inclusive education attitude.

*Hypothesis 2*: Empathy plays a mediating role between TM and inclusive education attitudes.

*Hypothesis 2a*: TM is significantly positively related to empathy.

*Hypothesis 2b*: Empathy is significantly positively related to inclusive education attitudes.

*Hypothesis 3*: Teacher efficacy for inclusive practice plays a mediating role between TM and inclusive education attitude.

*Hypothesis 3a*: TM is significantly positively related to teacher efficacy for inclusive practice.

*Hypothesis 3b*: Teacher efficacy for inclusive practice is significantly positively related to inclusive education attitude.

*Hypothesis 4*: Empathy is significantly positively related to teacher efficacy for inclusive practice.

*Hypothesis 5*: Empathy and inclusive educational efficacy play a sequential mediating role between TM and inclusive education attitudes.

**Figure 1:**
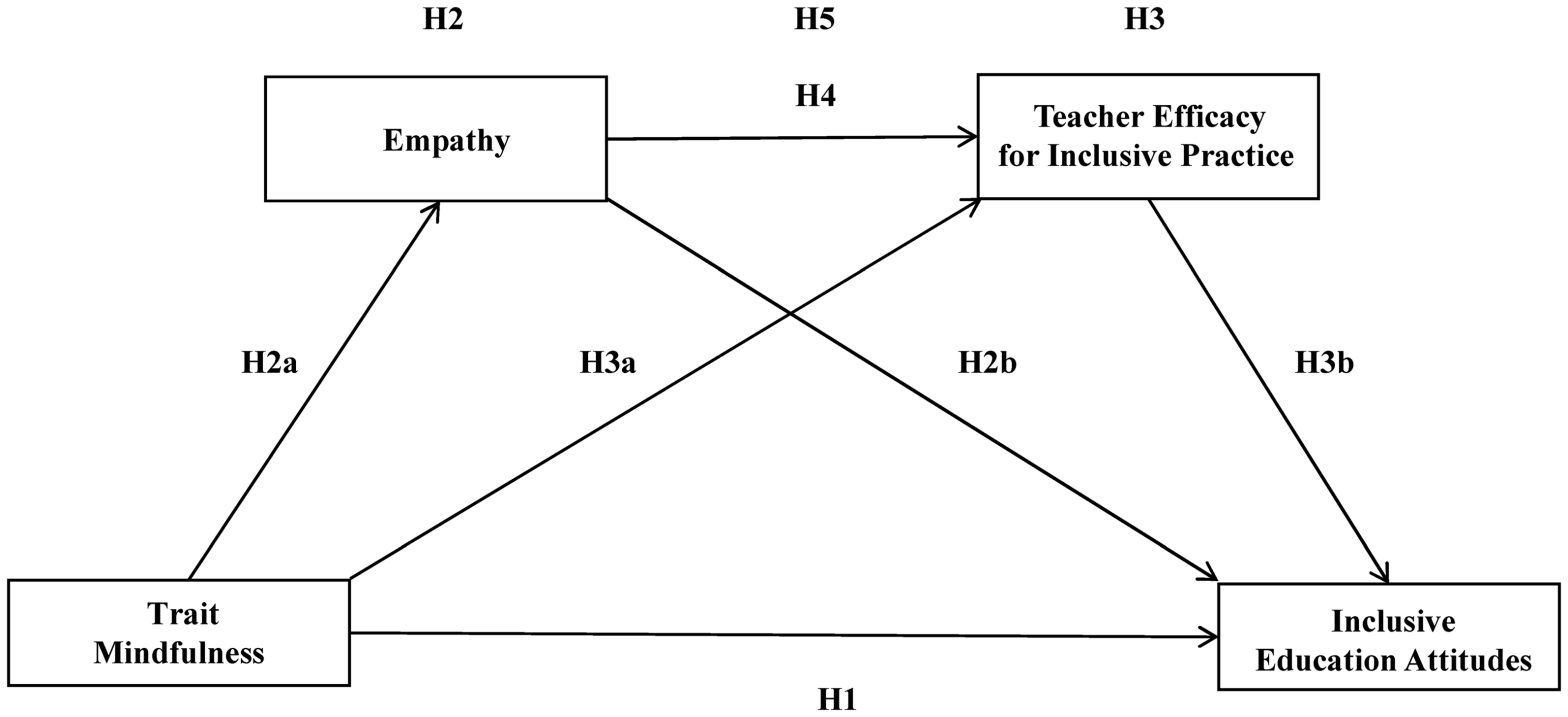
Theoretical hypothesis.

The diagram illustrating the theoretical hypothesis for this study is presented in Figure 1.

## 3 Methodology

### 3.1 Participants

In this investigation, a random sampling method was employed to select a cohort of 18 primary schools situated in Shandong Province, China. Data collection transpired from March 14 to March 21, 2023, encompassing responses from a total of 606 primary school teachers. The analytical dataset comprised 209 male primary school teachers and 397 female primary school teachers. Detailed demographic information, including gender, age, educational background, primary school location, and primary school nature, for all 606 subjects is presented in Table 1. The distribution of paper questionnaires occurred on-site, with a total of 678 questionnaires disseminated during the study. Of these, 623 questionnaires were returned. Following a meticulous review process that involved excluding questionnaires with evident errors and incomplete responses, a final tally of 606 valid questionnaires was achieved. The effective recovery rate for the questionnaires in this study was determined to be 89.38%. Moreover, it is imperative to note that the Research Ethics Committee of the corresponding author’s institution meticulously conducted a review and granted approval for research ethics, ensuring compliance with the principles outlined in the Declaration of Helsinki.

**Table 1:** Social demographic features of participants (*N* = 606).

Variables		Percentages
Gender	Male	34.49%
	Female	65.51%
Age	24–28 years old	73.10%
	29–33 years old	13.04%
	34–38 years old	7.92%
	39–43 years old	5.12%
	> 44 years old	0.83%
Educational Background	Junior college	3.14%
	Undergraduate	93.07%
	Postgraduate	3.80%
Primary School Location	Rural	33.33%
	Town	37.13%
	City	29.54%
Primary School Nature	Private	42.24%
	Public	57.76%

### 3.2 Measure

#### 3.2.1 Trait mindfulness

TM among elementary school teachers was measured using the Five Facet Mindfulness Questionnaire (Baer et al., 2006). Deng et al. (2011) translated the scale. This scale has been widely used in previous studies on the Chinese population (Dong et al., 2021). The scale consists of 39 items, including five dimensions of Observe, Describe, ActAware, NonJudge, and NonReact. An example question is, “I tell myself that I should not be feeling the way I’m feeling.” The scale uses Likert-5 scoring (1 = never or rarely, 5 = often or always). In the current survey data, the Cronbach’s α value of the scale was 0.972, and the Cronbach’s α value of the Observe, Describe, ActAware, NonJudge, and NonReact were 0.952, 0.936, 0.938, 0.921, and 0.927, respectively.

#### 3.2.2 Empathy

The Interpersonal Response Index is widely used to assess empathy. The scale was developed by Davis (1980), the Chinese version of the scale was translated and validated by Siu and Shek (2005). This scale has been widely used in previous studies in Chinese populations (Wang et al., 2023). The Chinese Interpersonal Response Index consists of three subscales: Fantasy, Empathetic Concern, and Personal Distress. There are 28 items in the scale. An example item such as, “I really get involved with the feelings of the characters in a novel.” This scale uses Likert-5 scoring (0 = does not describe me well, 4 = describes me well). In the current survey data, the total Cronbach’s α value of the Interpersonal Response Index was 0.930, and the Cronbach’s α value of the fantasy, empathetic concern, and personal distress were 0.841 0.902 and 0.905, respectively.

#### 3.2.3 Teacher self-efficacy for inclusive practice

The study used the Teacher Self-efficacy for Inclusive Practice Scale compiled by Sharma et al. (2012). The scale has good reliability and validity in China and has been widely applied (Malinen et al., 2012; Xie et al., 2022). The Teacher Self-efficacy for Inclusive Practice Scale consists of 18 items, including three factors: efficacy in using inclusive instruction (EUII), efficacy in collaboration (ECO), and efficacy in dealing with disruptive behaviors (EDDB). An example item is “I can set appropriate challenges for children with different abilities.” The scale uses a 6-point Likert scale (1 = strongly disagree, 6 = strongly agree). In the current survey data, the Cronbach’s α value of the scale is 0.924. The Cronbach’s α value of the three subscales were 0.859, 0.806 and 0.902, respectively.

#### 3.2.4 Inclusive education attitudes

The inclusive education attitudes adopts the Teachers’ Multidimensional Attitudes toward Inclusive Education Scale compiled by Mahat (2008). The Chinese version of the scale was translated and validated by Jia Chanjuan and Chunling (2020). The scale consists of 18 items and consists of three dimensions: Cognitive, Affective, and Behavioral. An example question is “I believe that students with a disability should be taught in special education schools.” The scale uses a 6-point Likert scale (1 = strongly agree, 6 = strongly disagree). In the current study, the Cronbach’s α value of the total is 0.894, and the Cronbach’s α value of the three subscales are 0.831, 0.815 and 0.852, respectively.

### 3.3 Data analysis method

This study uses SPSS 22.0 and Mplus 8.3 to analyze the data. We use SPSS 22.0 to input data and conduct demographic statistics and Pearson correlation analysis among variables. Mplus 8.3 is used for the fit test of the structural equation model and the test of the mediation path. In a structural equation model test, the primary school teacher’s gender, age, educational background, primary school location, and primary school nature of primary school teachers were used as control variables. In this research, we employed the Bootstrap method for data analysis. The core idea of this method is to generate multiple bootstrap samples by randomly sampling with replacement, allowing for the estimation of the distribution of a statistical measure through repeated sampling of these sets. To enhance the robustness and reliability of the results, I conducted 5,000 rounds of Bootstrap sampling.

## 4 Results

### 4.1 Common method Bias test

To mitigate the potential issue of method bias arising from the reliance on data sourced from a single report, a rigorous common method bias test was executed as a preliminary step. The Harman single factor test was selected as the methodological tool for this examination. The results revealed the presence of 15 factors with eigenvalues surpassing one, indicating a robust assessment of various dimensions within the data. Crucially, the cumulative variance explained by the first factor pair amounted to only 21.981%, well below the widely accepted threshold of 40% as advocated by Podsakoff et al. (2003). This outcome signifies that there is no significant common method bias concern impeding the validity of the study findings. The comprehensive application of the Harman single factor test, as outlined in this study, provides confidence in the integrity of the data analysis, affirming the absence of substantial methodological bias in the conducted research.

### 4.2 Descriptive statistical analysis

The correlation analysis was carried out on the scores of all facets of TM, empathy, teacher efficacy for inclusive practice, and inclusive education attitudes. The statistical results demonstrated that all facets of TM were significantly positively related to all facets of empathy, all facets of teacher efficacy for inclusive practice, and all facets of inclusive education attitudes. All facets of empathy were significantly positively correlated with all facets of Teacher efficacy for inclusive practice and all facets of inclusive education attitudes. All facets of teacher efficacy for inclusive practice and all facets of inclusive education attitudes were also significantly and positively correlated (Table 2).

**Table 2:** Means, standard deviations, and correlations of the major study variables.

Variable	M	SD	1	2	3	4	5	6	7	8	9	10	11	12	13	14	15	16	17	18	19
1.Gender	0.66	0.476	1																		
2.Age	28.580	4.511	−0.131**	1																	
3.Educational Background	2.010	0.263	−0.021	−0.368**	1																
4.Primary School Location	1.960	0.793	−0.039	0.039	0.207**	1															
5.Primary School Nature	1.580	0.494	0.054	−0.042	0.148**	0.360**	1														
6.Observe	3.413	0.917	−0.129**	0.250**	−0.061	0.159**	0.03	1													
7.Describe	3.518	0.800	−0.082*	0.179**	−0.074	0.173**	0.034	0.672**	1												
8.ActAware	3.430	0.829	−0.082*	0.176**	−0.061	0.220**	0.023	0.691**	0.631**	1											
9.NonJudge	3.284	0.781	−0.112**	0.211**	−0.079	0.171**	0.029	0.670**	0.654**	0.625**	1										
10.NonReact	3.167	0.894	−0.073	0.169**	−0.086*	0.156**	0.065	0.569**	0.567**	0.559**	0.535**	1									
11.FS	2.152	0.777	0.046	0.033	−0.014	0.042	0.068	0.161**	0.168**	0.154**	0.164**	0.220**	1								
12.EC	2.074	0.694	0.024	0.102*	−0.032	0.027	0.02	0.139**	0.144**	0.148**	0.169**	0.157**	0.420**	1							
13.PD	2.172	0.775	0.025	0.044	−0.027	0.051	0.042	0.170**	0.172**	0.188**	0.165**	0.181**	0.724**	0.388**	1						
14.EUII	3.739	0.910	−0.029	0.126**	−0.078	0.075	0.026	0.177**	0.186**	0.200**	0.165**	0.178**	0.232**	0.281**	0.268**	1					
15.ECO	3.454	0.853	−0.011	0.072	−0.045	0.092*	0.004	0.181**	0.190**	0.158**	0.183**	0.159**	0.149**	0.179**	0.244**	0.655**	1				
16.EDDB	3.660	1.035	−0.017	0.074	−0.085*	0.031	−0.022	0.121**	0.149**	0.159**	0.149**	0.142**	0.201**	0.190**	0.253**	0.594**	0.587**	1			
17.Cognitive	3.348	0.744	0.005	0.025	−0.010	0.063	0.004	0.236**	0.228**	0.214**	0.274**	0.164**	0.284**	0.299**	0.352**	0.404**	0.414**	0.371**	1		
18.Affective	3.245	0.816	0.018	0.092*	−0.077	0.061	0.017	0.236**	0.207**	0.198**	0.217**	0.190**	0.232**	0.273**	0.317**	0.353**	0.408**	0.355**	0.500**	1	
19.Behavioral	3.166	0.901	−0.015	0.077	−0.092*	−0.002	−0.022	0.137**	0.127**	0.117**	0.110**	0.134**	0.161**	0.223**	0.247**	0.328**	0.360**	0.282**	0.295**	0.643**	1

**p < 0.01, *p < 0.05. FS, Fantasy; EC, Empathic Concern; PD, Personal Distress; EUII, Efficacy in Using Inclusive Instruction; ECO, Efficacy in Collaboration; EDDB, Efficacy in Dealing with Disruptive Behaviors.

Gender is the dummy variable (0 = male, 1 = female).

### 4.3 Structural equation model index test

The Structural Equation Model (SEM) underwent testing utilizing Mplus software and the Maximum Likelihood (ML) method. The fit indices for the structural equation model within this study were as follows: ML *χ*^2^ = 305.51, degrees of freedom (df) = 126, *χ*^2^/df = 2.425, Comparative Fit Index (CFI) = 0.953, Tucker-Lewis Index (TFI) = 0.940, Root Mean Square Error of Approximation (RMSEA) = 0.048, and Standardized Root Mean Square Residual (SRMR) = 0.064. All seven indicators associated with the structural equation model demonstrated acceptability, as detailed in Table 3. These results collectively affirm the quality of the constructed model.

**Table 3:** Fit indices of the model.

Fit indices	Recommended threshold	Scores	Remarks
ML *χ*^2^	–	305.51	–
Df	–	126	–
*χ*^2^/df	1 < *χ*^2^/df < 3	2.425	Acceptable
CFI	> 0.9	0.953	Acceptable
TLI	> 0.9	0.940	Acceptable
RMSEA	< 0.08	0.048	Acceptable
SRMR	< 0.08	0.064	Acceptable

CFI, Comparative fit index; TLI, Tucker Lewis index; RMSEA, Root mean squared error of approximation; SRMR = Standardized root mean squared residual.

### 4.4 Test of mediation effect

We employed Mplus to examine the mediation model, and the results of structural equation modeling revealed significant path coefficients for TM, empathy, teacher efficacy for inclusive practice, and inclusive education attitudes.

Specifically, TM exhibited a positive relationship with inclusive education attitudes (β = 0.129, *p* = 0.005), supporting Hypothesis 1 posited in this study. Furthermore, TM demonstrated a positive association with empathy (β = 0.268, *p* < 0.001), endorsing Hypothesis 2a. Empathy, in turn, exhibited a positive correlation with inclusive education attitudes (β = 0.213, *p* < 0.001), substantiating Hypothesis 2b. Additionally, TM displayed a positive association with teacher efficacy for inclusive practice (β = 0.162, *p* = 0.001), confirming Hypothesis 3a. The relationship between teacher efficacy for inclusive practice and inclusive education attitudes was positive (β = 0.473, *p* < 0.001), supporting Hypothesis 3b. Lastly, empathy positively related to teacher efficacy for inclusive practice (β = 0.308, *p* < 0.001), corroborating Hypothesis 4. Refer to Table 4 for detailed results.

**Table 4:** The direct effect of the research paths and research model hypothesis analysis.

DV	IV	Std. Est.	S.E.	Est./S.E.	*p*-value	*R*^2^	Hypo and Path	Remarks
IEA	TM	0.129	0.046	2.818	0.005	0.405	H1: TM → IEA	Support
	Empathy	0.213	0.051	4.188	***		H2b: Empathy → IEA	Support
	TEIP	0.473	0.052	9.160	***		H3b: TEIP → IEA	Support
Empathy	TM	0.268	0.048	5.546	***	0.079	H2a: TM → Empathy	Support
TEIP	TM	0.162	0.050	3.229	0.001	0.156	H3a: TM → TEIP	Support
	Empathy	0.308	0.051	6.098	***		H4: Empathy → TEIP	Support

***p < 0.001.

IEA, Inclusive Education Attitudes; TM, Trait Mindfulness; TEIP, Teacher Efficacy for Inclusive Practice.

Table 5 delineates the indirect paths of structural equation modeling in this study. Empathy was identified as a mediator in the relationship between TM and inclusive education attitudes (β = 0.032, *p* = 0.005), with the 95% confidence interval for the path coefficient ranging from 0.014 to 0.059. This finding substantiates Hypothesis 2, and the mediating effect accounted for 19.16%.

**Table 5:** The indirect effect of the research paths.

Path	Std. Est.	S.E.	Est./S.E.	*p*-value	Boot LLCI	Boot ULCI	The proportion of the effect
H2: TM → Empathy → IEA	0.032	0.011	2.829	0.005	0.014	0.059	19.16%
H3: TM → TEIP → IEA	0.042	0.016	2.654	0.008	0.016	0.080	25.15%
H5: TM → Empathy → TEIP → IEA	0.022	0.007	2.905	0.004	0.010	0.040	13.17%
TOTALIND	0.096	0.026	3.665	***	0.050	0.152	57.49%
TOTAL	0.167	0.040	4.137	***	0.099	0.256	100.00%

***p < 0.001.

TM, Trait Mindfulness; IEA, Inclusive Education Attitudes; TEIP, Teacher Efficacy for Inclusive Practice.

Similarly, teacher efficacy for inclusive practice was identified as a mediator in the relationship between TM and inclusive education attitudes (β = 0.042, *p* = 0.008), with the 95% confidence interval for the path coefficient ranging from 0.016 to 0.080. This result supports Hypothesis 3, and the mediating effect accounted for 25.15%.

**Figure 2:**
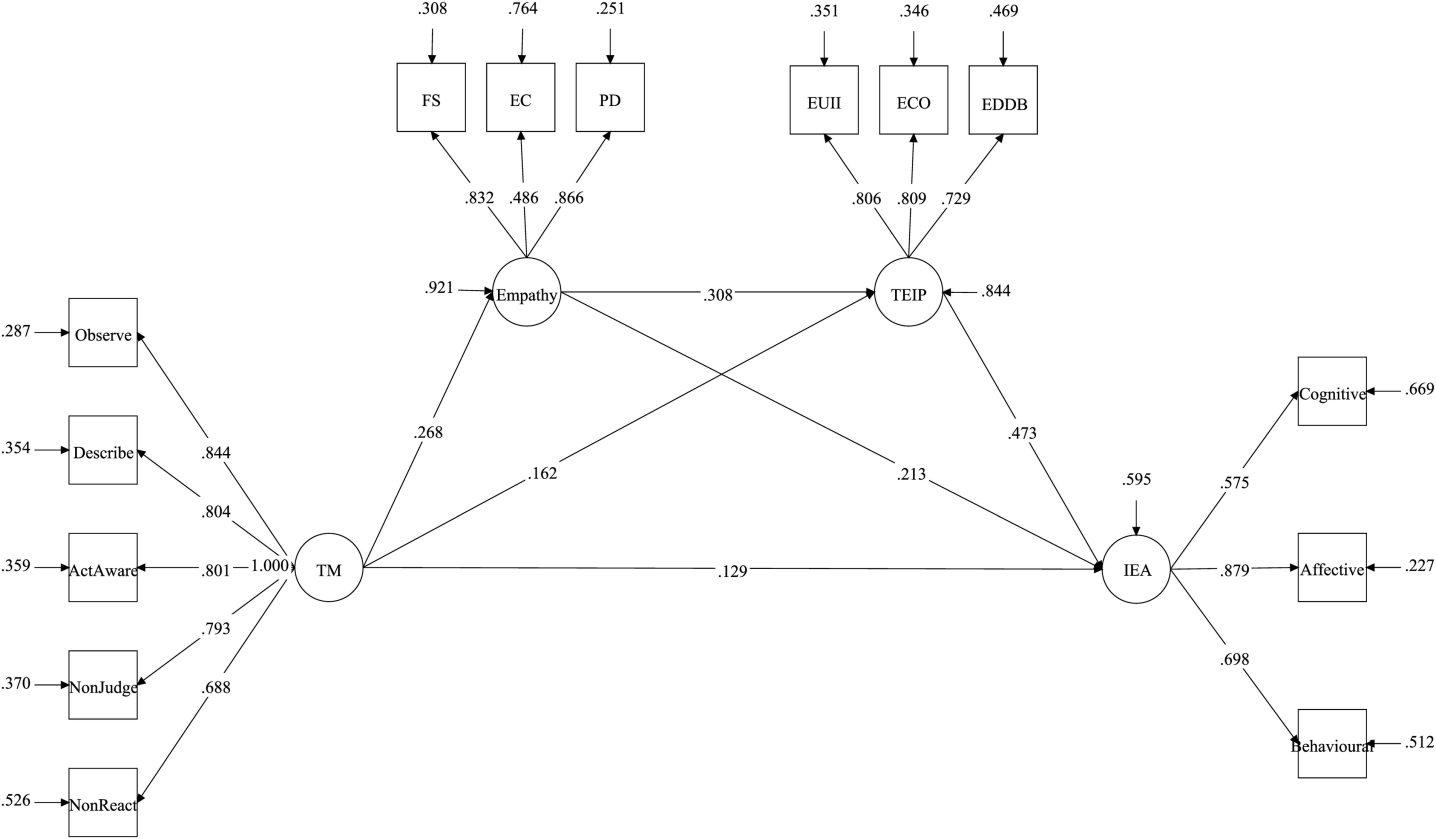
Structural equation model. TM, Trait mindfulness, TEIP, Teacher Efficacy for Inclusive Practice; IEA, Inclusive Education Attitudes; FS, Fantasy; EC, Empathic Concern; PD, Personal Distress; EUII, Efficacy in Using Inclusive Instruction; ECO, Efficacy in Collaboration; EDDB, Efficacy in Dealing with Disruptive Behaviors.

Furthermore, empathy and teacher efficacy for inclusive practice were found to sequentially mediate the relationship between TM and inclusive education attitudes (β = 0.022, *p* = 0.004), with the 95% confidence interval for the path coefficient ranging from 0.010 to 0.040. This outcome supports Hypothesis 5, with the mediating effect accounting for 13.17%. Refer to Figure 2 for a visual representation of these relationships.

## 5 Discussion

Based on the above research results, we found that the TM of primary school teachers can significantly and positively influence their inclusive education attitudes. This result validates our research hypothesis. TM can impact teachers’ caring abilities toward students (Kalafatoğlu and Turgut, 2019), reduce teachers’ tendency to be “self-centered,” contribute to improving teachers’ caregiving capabilities, and enhance the level of relationships between teachers and students. Additionally, TM can effectively alleviate teacher fatigue and exhaustion, enhance their ability to face challenges at work, and adequately address emotional needs in teaching (Lavy and Berkovich-Ohana, 2020). These beneficial qualities of TM have positive effects on the cognitive, emotional, and behavioral components of attitudes. Therefore, TM can significantly enhance the inclusive education attitudes of primary school teachers.

The results of this study indicate that empathy plays a mediating role between TM and inclusive education attitudes, with a mediation effect value of 19.16%. In other words, TM can influence the inclusive education attitudes of primary school teachers through empathy. This result confirms our research hypothesis. The effectiveness of mindfulness training in cultivating empathy has been supported by various studies (Hölzel et al., 2011; Bellosta Batalla et al., 2017). The attentional characteristics of mindfulness can help individuals recognize subtle emotional states in themselves and others, promoting understanding of others’ emotions (de la Fuente-Anuncibay et al., 2020). Additionally, at the emotional level, mindfulness contributes to cultivating an individual’s unconditional love, sympathy, and forgiveness towards others (Wu et al., 2019). Therefore, in the inclusive education system, primary school teachers with high empathy can profoundly understand the challenges and needs faced by special children from their perspective. This profound understanding often evokes feelings of compassion, leading teachers to care more about the children and be more willing to establish closer relationships with special children, thereby fostering a positive attitude towards inclusive education.

Additionally, this study found that the efficacy for inclusive practice of primary school teachers plays a mediating role between trait mindfulness and inclusive education attitudes, with a mediation effect value of 25.15%. In other words, TM can enhance the inclusive education attitudes of primary school teachers through their efficacy for inclusive practice. This result confirms our research hypothesis. Mindfulness is related to psychological empowerment and a sense of confidence (Perez-Blasco et al., 2013). This implies that individuals with high levels of mindfulness can generally better control their thoughts and emotions, thereby enhancing their self-efficacy. Teachers’ self-efficacy is crucial for teaching practices; it not only affects teaching effectiveness but also influences teachers’ efforts and professional identity (Martin and Mulvihill, 2019; Chen et al., 2020). Therefore, for primary school teachers involved in inclusive education, those with higher levels of mindfulness can better overcome challenges in their work, enhance self-efficacy, and ultimately have confidence in the successful implementation of inclusive education.

In addition to discovering that empathy and efficacy for inclusive practice play mediating roles in trait mindfulness and inclusive education attitudes, this study also found that empathy and efficacy for inclusive practice play a sequential mediating role in trait mindfulness and inclusive education attitudes. Teachers with empathy can proactively understand students’ individual situations and provide more care to students. Moreover, the more cognitive and emotional resonance teachers have with students, the more they can influence teachers’ perceptions of student behavior (Meyers et al., 2019). As Mercer et al. (2016) suggest, when teachers empathize with students, they strive to provide better education for them. Therefore, when teachers in inclusive education show empathy towards students with special needs, they gain a deeper understanding of the students’ situations and make ample teaching preparations. Thus, teachers with empathy often have stronger self-efficacy in their teaching.

This study provides practical insights for elementary school teachers involved in inclusive education. Firstly, schools and other educational institutions should actively organize mindfulness intervention training to enhance the mindfulness levels of elementary school teachers. On one hand, professionals can be hired to provide teachers with various types of mindfulness training programs, such as Mindfulness-based Cognitive Therapy and Mindfulness-based Stress Reduction. On the other hand, schools can incorporate mindfulness training into teacher training programs, helping teachers learn to manage and regulate their negative emotions, enhance their ability to cope with challenges, and build courage. Secondly, empathy training should be implemented to enhance teachers’ educational care. For example, experiential training and mindfulness training can be conducted to improve inclusive teachers’ interpretation skills of the behaviors of students with special needs, better meeting their needs in learning and daily life. Finally, to enhance teachers’ efficacy for inclusive practice, schools can establish integrated education professional training systems for teachers, helping them acquire relevant knowledge in special education and providing guidance for curriculum design and teaching development in inclusive education. Simultaneously, efforts should be made to strengthen teachers’ practical skills in inclusive education, such as regularly conducting group discussions, on-site observations, and case studies, helping teachers gain more practical experience to foster a more positive attitude towards inclusive education.

## 6 Limitations and future research directions

This study provides theoretical and practical guidance on how to improve the inclusive education attitudes of elementary school teachers. However, the study also has some limitations. Firstly, the teacher sample in this study was selected based on voluntary participation, which may have resulted in a selection bias, as these teachers might have had inherently positive attitudes toward inclusive education. However, measuring attitudes is a complex concept in itself. This is a questionnaire study and that teachers’ responses may be influenced by social desirability. Therefore, teachers’ actual attitudes and behaviors may be different in practice, and this should be observed in field studies. Secondly, while empathy is crucial for teachers and ensures the extent to which they provide support for students, some researchers have pointed out that if teachers’ empathetic behavior is not adequately supervised and if they overly prioritize students, it may lead to emotional exhaustion for teachers (Makoelle, 2019). Therefore, it cannot be assumed that stronger teacher empathy is always better. Finally, this study employed a cross-sectional design, which may not fully reflect the long-term effects of the variables. Future researchers could use longitudinal or intervention study approaches for a more in-depth measurement to examine whether these study results can be replicated.

## 7 Conclusion

The results of this study indicate a positive correlation between primary school teachers’ TM and their inclusive education attitudes. TM not only influences inclusive education attitudes through empathy but also affects inclusive education attitudes through efficacy for inclusive practice. Additionally, the study found that empathy and efficacy for inclusive practice play a sequential mediating role between primary school teachers’ TM and their inclusive education attitudes. This research not only enriches the research findings in the field of inclusive education for primary school teachers but also provides a theoretical foundation for studying the inclusive education attitudes of primary school teachers. Furthermore, the study offers a detailed explanation of the psychological mechanisms behind the impact of primary school teachers’ TM on inclusive education attitudes, providing direction for schools to implement mindfulness-based intervention programs.

## Data availability statement

The raw data supporting the conclusions of this article will be made available by the authors, without undue reservation.

## Ethics statement

The studies involving humans were approved by the Research Ethics Committee of Putian University. The studies were conducted in accordance with the local legislation and institutional requirements. The participants provided their written informed consent to participate in this study.

## Author contributions

FH: Conceptualization, Data curation, Formal analysis, Methodology, Writing – original draft.
